# Supplementary material for: Diet, Food Intake, and Exercise Mixed Interventions (DEMI) in the Enhancement of Wellbeing among Community-Dwelling Older Adults in Japan: Systematic Review and Meta-Analysis of Randomized Controlled Trials
Source: Geriatrics (Basel). 2024 Mar 4;9(2):32. doi: 10.3390/geriatrics9020032 (PMC10961817; doi:10.3390/geriatrics9020032)
Supplement: Supplementary file 1 [file geriatrics-09-00032-s001.zip › geriatrics-2835855-supplementary.pdf]

Supplementary Material

Table S1. Version 2 of the Cochrane risk-of-bias assessment tool for randomised trials: bias domains, signaling questions, response options, and risk-of-bias judgments

Bias domain and signalling question*	Seino (2017) [31]	Kwon (2015) [32]	Kawabata (2015) [33]	Takai (2013) [34]	Uemura (2018) [35]	Uemura (201~~9~~8) [36]	Sakurai (2012) [37]
Bias arising from the randomisation process<br>Risk-of-bias judgment (low/high/some concerns)<br>Optional: What is the predicted direction of bias arising from the randomisation process?	Low	Low	Low	Low	Low	Low	Some concerns
Bias due to deviations from intended interventions<br>Risk-of-bias judgment (low/high/some concerns)<br>Optional: What is the predicted direction of bias due to deviations from intended interventions?	Low	Low	Low	Low	Low	Low	Low
Bias due to missing outcome data<br>Risk-of-bias judgment (low/high/some concerns)<br>Optional: What is the predicted direction of bias due to missing outcome data?	Low	High	Low	Low	Low	Low	High
Bias in measurement of the outcome<br>Risk-of-bias judgment (low/high/some concerns)	Low	Low	Low	Low	Low	Low	Low

Optional: What is the predicted direction of bias in measurement of the outcome?							
Bias in selection of the reported result							
Risk-of-bias judgment (low/high/some concerns)	Low	Low	High	Low	Low	Low	Low
Optional: What is the predicted direction bias due to selection of the reported results?							
Overall bias							
Risk-of-bias judgment (low/high/some concerns)	Low	High	High	Low	Low	Low	High
Optional: What is the overall predicted direction of bias for this outcome?							

Y=yes; PY=probably yes; PN=probably no; N=no; NA=not applicable; NI=no information.

* Signalling questions for bias due to deviations from intended interventions relate to the effect of assignment to intervention.

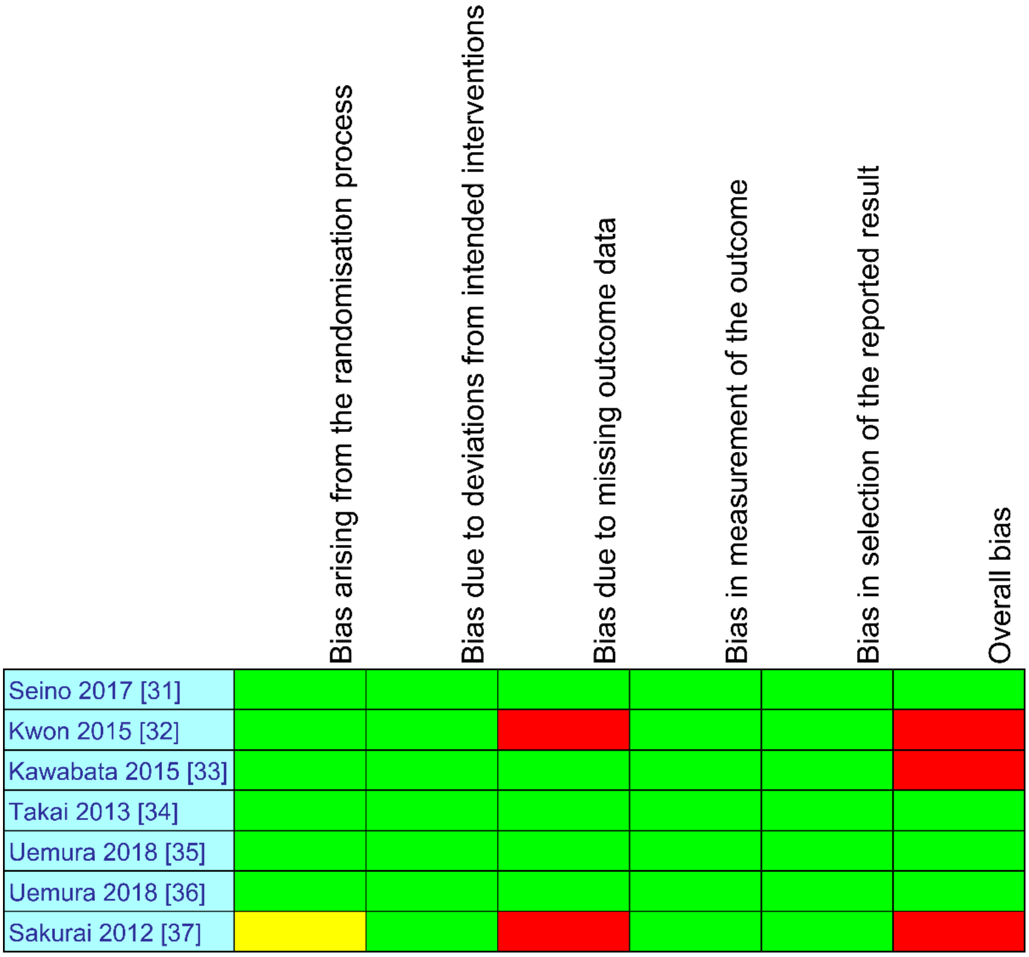

Figure S1. Risk of bias summary

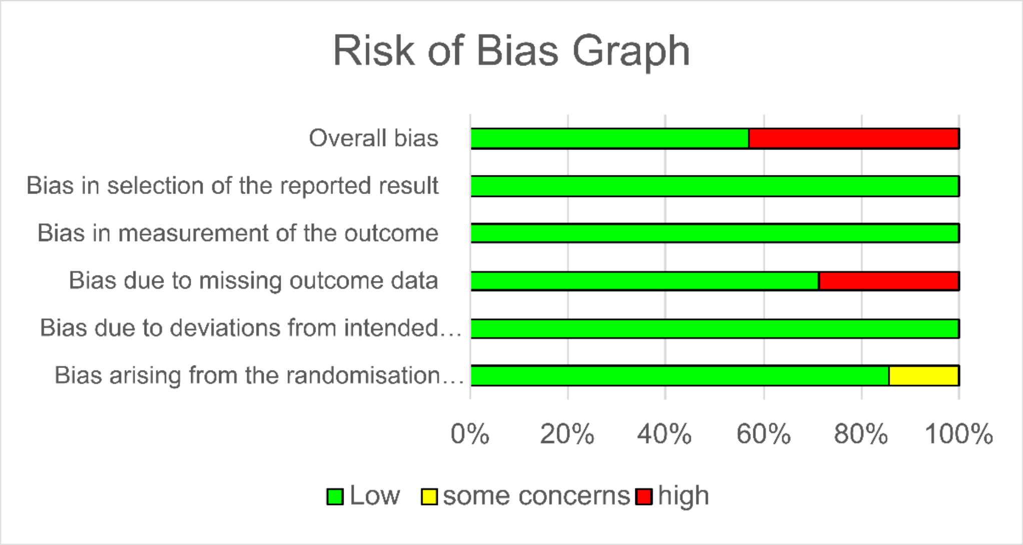

Figure S2. Risk of bias graph
